# Emergence of *Blastoschizomyces capitatus* Yeast Infections, Central Europe

**DOI:** 10.3201/eid1801.111192

**Published:** 2012-01

**Authors:** Tanja Birrenbach, Sonja Bertschy, Franziska Aebersold, Nicolas J. Mueller, Yvonne Achermann, Konrad Muehlethaler, Stefan Zimmerli

**Affiliations:** University Hospital Bern, Bern, Switzerland (T. Birrenbach, S. Zimmerli);; Kantonsspital Luzern, Lucern, Switzerland (S. Bertschy, F. Aebersold);; University Hospital Zurich, Zurich, Switzerland (N.J. Mueller, Y. Achermann);; University of Bern, Bern (K. Muehlethaler, S. Zimmerli)

**Keywords:** Blastoschizomyces capitatus, opportunistic fungal infection, fungi, immunosuppression, transplantation, Europe, Switzerland

## Abstract

We report 5 cases of disseminated infection caused by *Blastoschizomyces capitatus* yeast in central Switzerland. The emergence of this yeast in an area in which it is not known to be endemic should alert clinicians caring for immunocompromised patients outside the Mediterranean region to consider infections caused by unfamiliar fungal pathogens.

In recent years, the frequency of opportunistic fungal infections has increased in parallel with the growing number of patients receiving aggressive chemotherapy (*1*,*2*). Most fungal infections are caused by *Candida* spp. and *Aspergillus* spp., but reports of infections caused by rare molds and unusual yeasts are increasing. The widening range of fungal pathogens usually has been ascribed to the growing number of immunocompromised hosts and the use of antifungal prophylaxis (*1*,*3*). Another major contributor to the changing epidemiology of opportunistic fungal infections may be climatic changes (*4*,*5*), particularly for organisms for which geographic distribution appears to be restricted by climatic factors. We report the emergence of disseminated infections caused by *Blastoschizomyces capitatus* in the temperate zone of central Switzerland.

*B. capitatus*, a yeast formerly known as *Trichosporon* spp. and *Geotrichum capitatum*, can be isolated from the environment and may be a constituent of the microflora of the skin and the mucosa of the respiratory and digestive tracts (*6*). Colonization of human mucosa may precede invasion and hematogenous dissemination, resulting in invasive tissue infection of mainly the lung, liver, and skin (*7*–*9*). The dominant predisposing factor in >90% of reported cases is prolonged and profound neutropenia in patients with acute leukemia (*6*,*7*,*9*). Infections in neutropenic hosts share similarities with invasive candidiasis, but with *B. capitatus*, the rate of recovery from the bloodstream (≈80%), frequency of invasive tissue infections (≈60%), and rate of death (≈60%) are higher (*6*–*9*).

Climatic factors seem to play a selective role in the epidemiology of infections caused by *B. capitatus*. The geographic distribution of reported cases is restricted; most cases (87%) are observed in Europe, particularly in the Mediterranean region during the hottest period of the year. Italy, Spain, and France report 87% of the cases in Europe (*7*,*8*). Of the 99 *B. capitatus* infections reported worldwide through 2004, a total of 38 occurred in Italy (all but 1 in the central and southern regions), 30 in Spain, and 7 in France. Thus, approximately two thirds of all cases occurred in locations below 44° northern latitude, characterized by a Mediterranean climate with hot, dry summers and mild, wet winters. Central European countries, which have a temperate climate, contributed only 11 cases, and only 13 cases were reported from other continents (*7*). These findings are confirmed by recent data collected by the ARTEMIS DISK Surveillance Study, a longitudinal fungal surveillance program including *Candida* and non-*Candida* spp. yeasts isolated globally (*10*,*11*). To our knowledge, the only known case of invasive blastoschizomycosis in Switzerland was reported in 1983 (*12*).

## The Patients

Five patients with *B. capitatus* infections were observed at 3 tertiary care hospitals in Switzerland during June 2009–June 2011 (Table 1, Table 2). These hospitals provide services to a population of ≈3 million persons living in the temperate zone north of the Alps in an area ≈5,000 km^2^. A structured chart review was conducted for each patient, and further information was obtained from the involved infectious diseases specialist. The yeast was identified by using standard culture methods (Sabouraud dextrose agar with chloramphenicol and gentamicin and corn meal agar Tween 80; both from Oxoid, Cambridge, UK) and ID 32 C (bioMérieux, Marcy l’Etoile, France) (*13*). Identification of isolates was confirmed by DNA sequence analysis of the intergenic spacer (ITS) region (primers ITS1 + ITS4, GenBank accession nos. HQ014711.1 and HQ014712.1). For antifungal drug susceptibility testing, a microtiter broth dilution method based on the Clinical and Laboratory Standards Institute M27-A2 standard (Sensititre YeastOne; TREK Diagnostic Systems, East Grimstead, UK) was performed. Disk diffusion testing of fluconazole and voriconazole was performed as described (*10*). A review of the microbiology laboratory records of the 5 Swiss university centers yielded no additional isolations of *B. capitatus* in the past 5 years.

**Table 1:** Clinical characteristics of 5 patients with *Blastoschizomyces capitatus* yeast infection, Switzerland, June 2009–June 2010*

Patient no.	1	2	3	4	5
Age, y/sex	66/M	68/F	58/M	60/M	56/F
Underlying disease	AML	AML	AML	CAPD for renal failure after renal transplant	Diabetes mellitus, renal/pancreas transplant
Immunosuppression	Induction chemotherapy; reinduction 2×	Induction chemotherapy	Induction chemotherapy	CAPD; CycA, PDN	Tacrolimus, mycophenolate, PDN, thymoglobuline
Galactomannan assay	Negative	Negative	BAL positive; serum not done	Not done	Negative
Day of chemotherapy cycle at first isolation	14	13	22	NA	NA
Neutrophil count at diagnosis, × 10^9^ l^–1^	<0.1	<0.1	0.25	7.49	4.74
Days of neutropenia at diagnosis	16	8	18	NA	NA
Days of persistent fungemia/funguria	6	4	3	NA	114
Sites involved clinically	Lungs, skin, liver, spleen, brain	Lungs	Lungs, brain	Peritoneal fluid	Bladder, right kidney
Isolation of *B. capitatus*	Blood, skin	Blood	Blood, urine, DTA	Peritoneal fluid, peritoneal dialysis catheter, deep tissue biopsy	Urine
Sequential treatment after isolation (duration, d)	1. L-AMB (7)	1. L-AMB (2)	1. L-AMB (1)	1. VRC (28)	1. VRC (17)
	2. L-AMB + VRC (8)	2. VRC (3)	2. L-AMB + VRC (1)		2. FLC (8)
	3. L-AMB (25)		3. FLC (11)		3. AMB (4)
	4. VRC (145)				4. VRC (110, ongoing)
Days from collection of first positive sample to start of adequate treatment	0	1	3	4	3
Outcome (cause of death)	Death 185 d after diagnosis (relapsing leukemia and MOF)	Death 9 d after diagnosis (MOF)	Death 15 d after diagnosis (MOF)	Alive	Alive
Contribution of *B. capitatus* to death	–	+	+	NA	NA
Travel history during previous year	Trip to southern France 3 months before diagnosis	None	Unknown	None	None

*Diagnosis is defined as the day of collection of first sample positive for *B. capitatus.* No patients received antifungal prophylaxis. AML, acute myeloid leukemia; CAPD, continuous ambulatory peritoneal dialysis; CycA, cyclosporine A; PDN, prednisone; BAL, bronchoalveolar lavage; NA, not applicable; DTA, deep tracheal aspiration; L-AMB, liposomal amphotericin B; VRC, voriconazole; FLC, fluconazole; AMB, deoxycholate amphotericin B; MOF, multiorgan failure.

**Table 2:** In vitro antifungal susceptibility profile according to microtiter broth dilution for patients with *Blastoschizomyces capitatus* yeast infections, Switzerland, June 2009–June 2010*

Patient no.	MIC, μg/mL						
	AMB	5-FC	FLC	ITC	VRC	POS	CAS
1	0.5	0.03	2	0.06	0.016	0.25	4
2	0.5	0.03	8	0.12	0.06	0.25	4
3	0.5 (*3*)†	0.06	4	0.06	0.06	0.25	8
4	0.5	16	1	0.032	0.032	0.064	4
5	0.5	0.12	16	0.25	0.25	0.5	>16

*AMB, deoxycholate amphotericin B; 5-FC, 5-fluorocytosine; FLC, fluconazole; ITC, itraconazole; VRC, voriconazole; POS, posaconazole; CAS, caspofungin. †By Etest (bioMérieux, Marcy l’Etoile, France), the isolate had an AMB MIC of 3 μg/mL.

Patient 1 was a 66-year-old man with secondary acute myeloid basophilic leukemia. He became febrile, and pulmonary nodules and pustular skin lesions developed after induction chemotherapy. Blood cultures and cultures from a skin lesion yielded *B. capitatus*. The patient was treated with amphotericin B and voriconazole, both of which had low MICs. Skin lesions improved within days, and pulmonary nodules resolved within weeks. Eventually, relapsing leukemia proved refractory, and the patient died of multiorgan failure.

Patient 2 was a 68-year-old woman with acute myeloid leukemia. Neutropenic fever developed after induction chemotherapy. Blood cultures yielded *B. capitatus*. She was treated with liposomal amphotericin B and voriconazole. However, she died of multiorgan failure 9 days after diagnosis.

Patient 3 was a 58-year-old man receiving induction chemotherapy for acute myeloid leukemia, who was treated for polybacterial neutropenic sepsis. Caspofungin was added because of persistent fever. *B. capitatus* was isolated from blood cultures, urine, and tracheobronchial fluid while he was being treated. Antifungal therapy was switched to liposomal amphotericin B and voriconazole. When a preliminary report indicated an amphotericin B MIC >2 µg/mL (Etest; bioMérieux) for the isolate, treatment was changed to fluconazole. Multiorgan failure occurred, and he died 15 days after diagnosis. Autopsy findings confirmed extensive fungal pulmonary infiltrates with angioinvasion and multiple foci of *B. capitatus* infection in the liver, spleen, kidneys, bone marrow, myocardium, and brain.

Patient 4 was a 60-year-old man who was receiving continuous ambulatory peritoneal dialysis for renal failure 15 years after kidney transplantation for chronic glomerulonephritis; he sought care for acute peritonitis. Upon cultivation of yeasts in the dialysis fluid, caspofungin was started and changed to voriconazole when *B. capitatus* was identified. The patient’s symptoms rapidly improved, and voriconazole was discontinued after 4 weeks.

Patient 5 was a 56-year-old woman who underwent combined pancreas–kidney transplantation because of diabetic nephropathy and pancreatic insufficiency in December 2010. Two months later, *B. capitatus* was cultivated in urine while she was being treated empirically with caspofungin for possible fungal infection. Emphysematous cystitis was detected by computed tomographic scan, and *B. capitatus* was cultured from urine collected from the right kidney. Despite treatment with voriconazole, *B. capitatus* was cultured from urine for several weeks; however, the patient recovered.

## Conclusions

The repeated isolation of *B. capitatus* in a temperate climate zone was unexpected because of the well-documented restriction of *B. capitatus* to areas with a Mediterranean climate >450 km south in Italy, Spain, and France. Because of the high recovery rate of the organism from blood cultures of patients with disseminated infection and the ease of culturing *B. capitatus* on standard media, we believe it unlikely that a large number of cases could have been missed in the past.

Central Switzerland has witnessed a steady rise in temperatures since the mid-1980s. Mean annual temperatures for the past 25 years were all above the mean for 1961–1990 and now exceed those of 1980 by >1°C. The rise in average temperatures was more pronounced during spring and summer months. Since 1981, average spring and summer temperatures in central Switzerland have increased by 0.77°C and 0.48°C per decade, respectively (*14*).

The occurrence of 4 of the 5 infections reported here during the warm season indicates that the rising temperatures might have contributed to the expanded range of climatically restricted fungi to cooler areas and that the emergence of *B. capitatus* might be a consequence of the local effects of global warming (*5*,*15*). Alternatively, importation of the pathogen from areas to which it is endemic through increasing traffic of humans and goods might have led to establishment of a new endemic hotspot. Our observation should alert clinicians caring for severely immunocompromised patients in temperate areas to consider infections caused by unfamiliar fungal pathogens, notably *B. capitatus*.
